# Obinutuzumab treatment for antineutrophil cytoplasmic antibody-associated vasculitis

**DOI:** 10.3389/fimmu.2026.1787558

**Published:** 2026-05-13

**Authors:** Chen Ye, Zhi-ying Li, Xiang-yu Han, Ming-hui Zhao, Min Chen

**Affiliations:** 1 Renal Division, Department of Medicine, Peking University First Hospital, Beijing, China; 2 Institute of Nephrology, Peking University, Beijing, China; 3 Key Laboratory of Renal Disease, Ministry of Health of China, Beijing, China; 4 Key Laboratory of Chronic Kidney Disease Prevention and Treatment (Peking University), Ministry of Education, Beijing, China; 5 Research Units of Diagnosis and Treatment of Immune-mediated Kidney Diseases, Chinese Academy of Medical Sciences, Beijing, China; 6 Department of Nephrology, Union Hospital, Tongji Medical College, Huazhong University of Science and Technology, Wuhan, China; 7 State Key Laboratory of Vascular Homeostasis and Remodeling, Peking University, Beijing, China

**Keywords:** antineutrophil cytoplasmic antibody, B-cell, immunotherapy, obinutuzumab, vasculitis

## Abstract

**Background:**

Anti-CD20 therapy with rituximab serves as the cornerstone of both induction and maintenance treatment for antineutrophil cytoplasmic antibody (ANCA)-associated vasculitis (AAV). Obinutuzumab, a humanized type II anti-CD20 monoclonal antibody, is designed for more potent and sustained B-cell depletion with improved tolerability. Due to the paucity of clinical evidence of obinutuzumab in AAV, we undertook this study to assess its efficacy and safety profile.

**Methods:**

Twelve consecutive patients with AAV who received first obinutuzumab treatment between September 2022 and December 2024 were included in this retrospective study. Clinical and pathological data were collected at predefined time points.

**Results:**

Four patients received obinutuzumab for induction therapy, and 8 for maintenance therapy. The median follow-up duration after obinutuzumab administration was 17.5 months. Among the 4 patients receiving obinutuzumab for induction therapy, one patient progressed to end-stage kidney disease after infection, while the other 3 achieved sustained remission. Among the 8 patients receiving obinutuzumab for maintenance therapy, no relapse occurred. Among all the patients, three experienced at least one severe adverse event following obinutuzumab therapy. B-cell reconstitution typically began 9–12 months after obinutuzumab administration, with one patient showing no reconstitution at 24 months of follow-up. Concomitant with B-cell depletion, ANCA levels gradually declined in most patients and became undetectable in half. Notably, ANCA levels continued to decrease for up to one year after obinutuzumab administration, even after B-cell reconstitution began.

**Conclusion:**

Obinutuzumab was effective and well-tolerated in AAV patients, supporting its potential as an alternative anti-CD20 therapy for AAV.

## 1 Introduction

Antineutrophil cytoplasmic antibody (ANCA)-associated vasculitis (AAV) comprises a group of severe autoimmune diseases, including granulomatosis with polyangiitis (GPA), microscopic polyangiitis (MPA) and eosinophilic granulomatosis with polyangiitis (EGPA). AAV is characterized as a systemic small-vessel vasculitis, accompanied by the presence of circulating ANCAs that target cytoplasmic constituents of neutrophils and monocytes, especially myeloperoxidase (MPO) or proteinase 3 (PR3) (1).

ANCAs induce excessive neutrophil activation, leading to small blood vessel injury. However, other immune cells, including dendritic cells, macrophages, B cells and T cells, and the complement system are also involved in the pathogenesis of AAV. Among them, B cells play a pivotal role in AAV development, with their functions extending beyond serving as precursors for ANCA-producing plasma cells. As professional antigen-presenting cells, B cells efficiently present antigens to autoreactive T cells and provide co-stimulatory signals necessary for T cell activation. Additionally, B cells can secrete pro-inflammatory cytokines such as interleukin-6 (IL-6) and tumor necrosis factor (TNF), which can diminish the anti-inflammatory effects of regulatory T (Treg) cells and promote the differentiation of effector T cells (2, 3). Furthermore, the association between B-cell activation and disease activity, together with the clinical efficacy of B-cell depletion therapy, strongly supports a pivotal role for B cells in AAV pathogenesis (2).

Over the past decades, rituximab, a chimeric anti-CD20 monoclonal antibody (mAb) for B-cell depletion, has been well-established as induction and maintenance treatment for AAV (2). However, optimal disease control is not achieved in all patients. This may be partly attributed to incomplete depletion of tissue-resident B-cell subsets and limited tissue penetration (4, 5). Additionally, the internalization and trogocytic removal of rituximab-CD20 complexes may further compromise therapeutic efficacy (6). Obinutuzumab, a humanized type II anti-CD20 antibody, has been shown to induce enhanced direct B-cell cytotoxicity and may facilitate more effective depletion of B cells, including tissue-resident populations, with less susceptibility to internalization and trogocytic clearance (6–8). These properties provide a biological rationale for its potential use as an alternative anti-CD20 therapy. In clinical practice, obinutuzumab has shown favorable efficacy in the treatment for hematological malignancies and primary membranous nephropathy (9, 10). However, to our knowledge, data on the efficacy and safety of obinutuzumab in AAV remain limited, with only several small case series reported (11–14). Herein, we presented our single-center retrospective observational experience with obinutuzumab for the treatment of AAV.

## 2 Materials and methods

### 2.1 Patients and therapy

AAV patients who received first obinutuzumab treatment between September 2022 and December 2024 at Peking University First Hospital were retrospectively recruited. All the patients met the Chapel Hill Consensus Conference nomenclature criteria for AAV (15). Patients with EGPA, secondary vasculitis, or other coexisting renal diseases, as well as younger than 14 years, were excluded. This retrospective, non-interventional observational study was conducted in accordance with the Declaration of Helsinki and was approved by the Ethics Committee of Peking University First Hospital.

A single fixed dose of 1000 mg obinutuzumab was given as induction therapy for the treatment of newly diagnosed or relapsing AAV. In the absence of an established evidence-based dosing regimen for obinutuzumab in AAV, and considering the infection risk associated with intensive immunosuppressive therapy, this approach was adopted as an exploratory strategy. For maintenance therapy, obinutuzumab (1000 mg) was administered either as initial maintenance or as an alternative regimens, with re-dosing triggered when CD19^+^ B-cell count exceed 5 cells/μL in peripheral blood, adapted from the tailored rituximab-based strategy (16), or, based on clinical practice, approximately every 9–12 months. The counts of CD19^+^ B lymphocytes and ANCA levels were assessed every 2–3 months. Glucocorticoids were tapered or withdrawn accordingly. Obinutuzumab was obtained by local hospital pharmacies for off-label use. All patients signed an informed consent. All patients were followed up for at least 6 months after obinutuzumab administration.

### 2.2 Data collection and disease assessment

Data on patient demographics, clinical manifestations, renal pathology, treatment strategies, and outcomes were collected. Estimated glomerular filtration rate (eGFR) was calculated using the Chronic Kidney Disease Epidemiology collaboration (CKD-EPI) creatinine equation (17). Disease activity was measured by the Birmingham Vasculitis Activity Score (BVAS) (18). Remission was defined as a BVAS of 0, and response as a > 50% reduction in BVAS and absence of new manifestations of active vasculitis. Relapses were defined as re-occurrence or new onset of disease attributable to active vasculitis (19). End-stage kidney disease (ESKD) was defined as the need for maintenance dialysis or kidney transplantation. Information about severe adverse events was also recorded.

### 2.3 Renal histology

The histopathological assessment was performed according to the previously standardized protocol for scoring renal biopsies in patients with AAV and the Berden classification (20, 21). Glomerular lesions were calculated as the percentage of the total number of glomeruli. The Berden classification composed of four classes: focal, ≥ 50% normal glomeruli; crescentic, ≥ 50% glomeruli with cellular crescents; sclerotic, ≥ 50% globally sclerotic glomeruli; and mixed, < 50% normal, < 50% crescentic, < 50% globally sclerotic glomeruli (21). Interstitial fibrosis and tubular atrophy (IFTA) was scored semi-quantitatively according to the proportion of the affected tubulointerstitial compartment: 0, absent; 1+, < 25%; 2+, 25–50% and 3+, > 50% of the total area, as previously described (20).

### 2.4 Statistical analysis

Continuous variables were presented as median (interquartile range, IQR) or mean ± standard deviation (SD), as appropriate. Categorical variables are expressed as counts (percentage). Data analyses were performed with SPSS (version 25.0, IBM Corp, Armonk, NY).

## 3 Results

### 3.1 Patient characteristics

Twelve patients with AAV treated with obinutuzumab for the first time between September 2022 and December 2024 were retrospectively recruited. Baseline characteristics of these patients at the time of diagnosis were summarized in **Table 1**. Among these 12 patients, 6 were male and 6 were female, with a median age of 50 (range, 18–73; IQR, 32.25–62.75) years at diagnosis. One patient (Patient 5) was diagnosed with PR3-AAV in the setting of disease relapse, whereas all others were newly diagnosed MPO-AAV. All patients had renal involvement, and 4 patients had pulmonary involvement. The median levels of serum creatinine (Scr) and eGFR at presentation were 183.00 μmol/L (IQR, 106.00–566.53) and 29 mL/min/1.73m^2^ (IQR, 8–55), respectively. Eight patients underwent renal biopsy. According to the Berden classification, 4 were classified as mixed class, 3 as focal class, and 1 as crescentic class. The median proportion of normal glomeruli, cellular crescents, and fibrous crescents was 41.41% (IQR, 17.40–64.48), 38.59% (IQR, 24.11–44.62) and 0.00% (IQR, 0.00–7.14), respectively.

**Table 1:** Baseline characteristics of patients with AAV at diagnosis.

Patient	Sex	Age (years)	Disease status	ANCA subtypes	Organ involvement	Proteinuria (g/d)	Scr (μmol/L)	eGFR (mL/min/1.73 m^2^)	Berden classification	Normal glomeruli (%)	Cellular crescents (%)	Fibrous crescents (%)	IFTA
1	M	64	Newly diagnosed	MPO-ANCA	kidney	NA	118.60	56	Mixed	0	23.81	9.52	1+
2	F	51	Newly diagnosed	MPO-ANCA	kidney	0.71	178.00	28	Focal	57.89	42.11	0	2+
3	M	73	Newly diagnosed	MPO-ANCA	kidney, lung, nervous system	NA	669.00	6					
4	M	40	Newly diagnosed	MPO-ANCA	kidney	3.86	736.40	7					
5	M	18	Relapsing	PR3-ANCA	kidney	2.18	101.80	92	Crescentic	36.67	63.33	0	1+
6	F	24	Newly diagnosed	MPO-ANCA	kidney	NA	524.00	9	Mixed	21.21	45.45	21.21	0
7	F	39	Newly diagnosed	MPO-ANCA	kidney, skin	3.39	306.80	16	Focal	67.39	21.74	0	2+
8	F	56	Newly diagnosed	MPO-ANCA	kidney	NA	101.70	53					
9	M	70	Newly diagnosed	MPO-ANCA	kidney, lung	1.78	101.22	64	Focal	66.67	25.00	0	0
10	F	30	Newly diagnosed	MPO-ANCA	kidney, lung, ENT, eyes	2.67	188.00	30	Mixed	46.15	38.46	0	2+
11	F	49	Newly diagnosed	MPO-ANCA	kidney, lung	0.66	580.70	7	Mixed	16.13	38.71	0	2+
12	M	59	Newly diagnosed	MPO-ANCA	kidney	NA	175.00	36					

ANCA, antineutrophil cytoplasmic antibody; eGFR, estimated glomerular filtration rate; ENT, Ear, Nose, and Throat; F, female; IFTA, interstitial fibrosis and tubular atrophy; M, male; MPO, myeloperoxidase; NA: not available; PR3, proteinase 3; Scr, serum creatinine.

### 3.2 Obinutuzumab therapy and outcomes

The median time from AAV diagnosis to the initiation of obinutuzumab was 12.5 months for all patients and 0.5 month in the induction treatment subgroup. Characteristics of these patients at obinutuzumab administration and clinical outcomes were shown in **Table 2**. The levels of Scr and eGFR at obinutuzumab administration were 122.03 (IQR, 103.25–254.25) μmol/L and 46 (IQR, 19–65) mL/min/1.73m^2^, respectively. Proteinuria ranged from negative to 2+, with one patient having nephrotic-range proteinuria (3.86 g/24 h).

**Table 2:** Data and clinical outcomes of patients with AAV during obinutuzumab therapy.

Patient	Time from diagnosis of AAV to obinutuzumab administration (months)	Treatment setting of obinutuzumab	Prior induction therapy	Prior maintenance therapy	Prior cumulative dose of rituximab (mg)	At obinutuzumab administration				Cumulative dose of obinutuzumab (mg)	At the 12-month follow-up			At last visit		Relapse	SAE	Follow-up time after obinutuzumab administration (months)
						ANCA status	Proteinuria	Scr (μmol/L)	eGFR (mL/min/ 1.73 m^2^)		Scr (μmol/L)	eGFR (mL/min/ 1.73 m^2^)		Scr (μmol/L)	eGFR (mL/min/ 1.73 m^2^)			
1	4	Induction	P, RTX	-	1000	Positive	2+	103.00	66	1000	98.32	69		112.00	59	No	No	17
2	0.5	Induction	P	-	0	Positive	2+ (0.71 g/24 h)	104.00	54	1000	92.00	62		80.85	71	No	No	39
3	0.5	Induction	P, PE	-	0	Positive	Trace	419.00	11	1000	189.33	30		196.63	28	No	Pneumonia (twice)	31
4	0.5	Induction	P	-	0	Positive	3+ (3.86 g/24 h)	602.00	9	1000	HD	HD		HD	HD	N/A	Pneumonia	13
5	9	Initial maintenance	P, CTX, RTX	-	1500	Negative	2+ (0.59 g/24 h)	96.48	98	2000	91.44	104		88.99	107	No	No	15
6	16	Initial maintenance	P, RTX, PE	-	3500	Positive	NA	231.00	25	2000	NA	NA		289.19	19	No	No	9
7	9	Initial maintenance	P, RTX	-	1500	Positive	2+	78.64	81	1000	61.38	108		66.56	99	No	No	13
8	25	Alternative maintenance	P, CTX	RTX	1300	Positive	2+	134.00	38	2000	119.10	43		95.23	56	No	No	24
9	34	Alternative maintenance	P, RTX	RTX	3500	Negative	Negative	110.05	57	3000	88.70	74		94.62	68	No	No	18
10	47	Alternative maintenance	P, RTX	AZA/MTX	2400	Positive	2+	106.10	60	2000	108.09	59		118.37	52	No	No	23
11	47	Alternative maintenance	P, CTX, PE	RTX	2500	Negative	1+	262.00	17	2000	284.32	16		355.40	12	No	No	23
12	31	Alternative maintenance	P, CTX	RTX/AZA/ MMF	500	Negative	Trace	164.70	38	2000	139.63	47		144.65	45	No	Pneumonia	15

AAV, antineutrophil cytoplasmic antibody-associated vasculitis; ANCA, antineutrophil cytoplasmic antibody; AZA, azathioprine; CTX, cyclophosphamide; eGFR, estimated glomerular filtration rate; HD, hemodialysis; MMF, mycophenolate mofetil; MTX, methotrexate; NA: not available; N/A: not applicable; PE, plasma exchange; P, prednisone; RTX, rituximab; SAE, severe adverse event; Scr, serum creatinine.

Four patients (Patients 1–4) received glucocorticoids and obinutuzumab as induction therapy. Among them, Patient 1 had a cumulative dose of 1000 mg rituximab prior to obinutuzumab, while Patient 2 received 600 mg rituximab after obinutuzumab. Patient 4 received a dose of 0.4 g cyclophosphamide combined with obinutuzumab. Patient 3 received temporary dialysis and plasma exchange shortly after initial presentation.

Eight patients (Patients 5–12) received obinutuzumab as maintenance therapy, including 3 patients as initial maintenance and 5 patients as alternative maintenance regimens. Prior to obinutuzumab administration, all had received glucocorticoids and rituximab, with a mean cumulative dose of rituximab of 2087.5 ± 1075.0 mg. Additionally, before obinutuzumab administrations, 4 patients had received cyclophosphamide during induction therapy, while 2 patients had taken oral azathioprine, methotrexate or mycophenolate mofetil for maintenance therapy. Patients 6 and 11 received temporary dialysis and plasma exchange shortly after initial presentation.

The median duration of follow-up after obinutuzumab administration was 17.5 (range, 9–39; IQR, 13.5–23.8) months. Among the 4 patients who received obinutuzumab for induction therapy, 3 achieved sustained remission during the follow-up period, while 1 (Patient 4) progressed to ESKD 4 months after infusion due to COVID-19-associated renal deterioration. In the 8 patients who received obinutuzumab for maintenance therapy, no relapse occurred during the follow-up period. At the last follow-up, among these 12 patients, only Patient 4 remained on maintenance dialysis. In the remaining 11 patients, the median Scr level was 112.00 μmol/L (IQR, 88.99–196.63) and eGFR was 56 mL/min/1.73m^2^ (IQR, 28–71). To further characterize changes in renal function over time, we performed a paired analysis at 12 months. After excluding Patient 4 (who initiated dialysis) and Patient 6 (who was lost to follow-up at 9 months), the remaining 10 patients had an eGFR of 61 ± 29 mL/min/1.73 m² at 12 months, compared with 52 ± 27 mL/min/1.73 m² at obinutuzumab initiation. This difference was statistically significant in the paired analysis (*P* = 0.011).

### 3.3 B-cell depletion and ANCA dynamics

**Figure 1:**
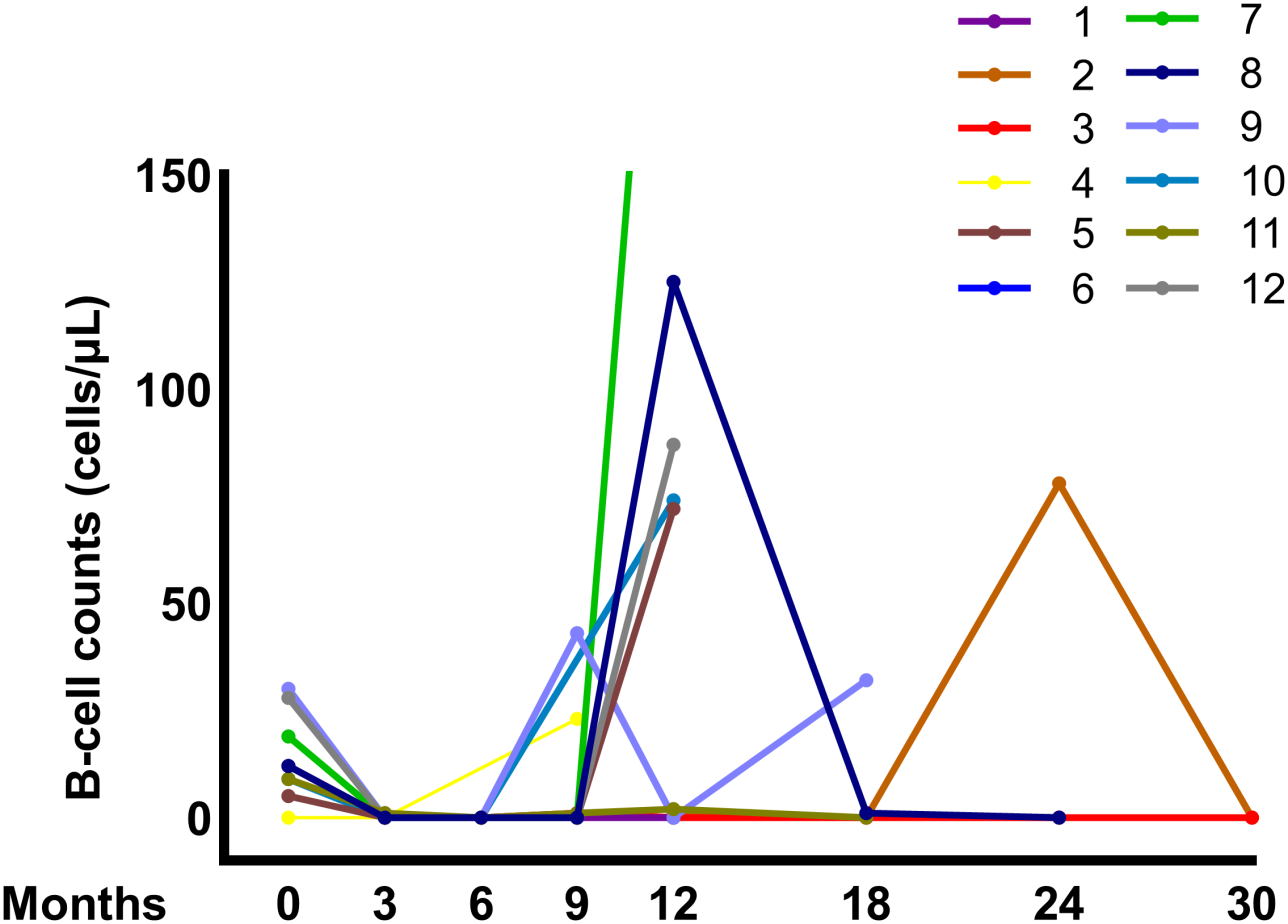
B-cell dynamics after obinutuzumab administration.

Longitudinal B-cell counts and ANCA levels were shown in **Figure 1**, with individual patient data presented in **Supplementary Table 1** and **Table 3**. The median B-cell count was 12.00 (IQR, 7.00–23.50) cells/μL before obinutuzumab administration. By 3 months after obinutuzumab administration, 90% (9/10) of tested patients had complete B-cell depletion (0 cells/μL), with one patient maintaining a residual count of 1 cell/μL. By 6 months after obinutuzumab administration, all tested patients achieved complete B-cell depletion. B-cell reconstitution (> 5 cells/μL) occurred in 2 patients (Patients 4 and 9) at 9 months after obinutuzumab administration, and in another 5 patients (Patients 5, 7, 8, 10 and 12) at 12 months after obinutuzumab administration. In Patients 2 and 3, B-cell reconstitution was delayed until 24 months after obinutuzumab administration and beyond. Among these patients, Patients 5, 8 and 12 received an additional dose of obinutuzumab, while Patient 9 received two additional doses. Patient 7 switched from obinutuzumab to rituximab, as obinutuzumab was not readily accessible. Patient 10 temporarily received alternative therapy during pregnancy, with re-dosing deferred until the postpartum period. Patient 4 stopped immunosuppressive treatment after progression to ESKD. During maintenance, four patients (Patients 8, 9, 11, and 12) switched from rituximab to obinutuzumab owing to early B-cell reconstitution. In these patients, dosing intervals increased from 3–6 to 9–14 months after switching, generally in accordance with CD19^+^ B-cell reconstitution, suggesting more prolonged B-cell depletion compared with prior rituximab treatment.

**Table 3:** ANCA dynamics after obinutuzumab administration.

Patient	ANCA subtype	ANCA levels (RU/ml)							
		Baseline	Month 3	Month 6	Month 9	Month 12	Month 18	Month 24	Month 30
1	MPO-ANCA	42	Negative	–	Negative	Negative	–	–	–
2	MPO-ANCA	Positive	–	–	171	184	>200	>200	>200
3	MPO-ANCA	>200	Negative	Negative	Negative	Negative	Negative	Negative	Negative
4	MPO-ANCA	111.83	Negative	Negative	–	–	–	–	–
5	PR3-ANCA	Negative	Negative	Negative	Negative	–	–	–	–
6	MPO-ANCA	58.6	–	–	37	–	–	–	–
7	MPO-ANCA	137	95	54	56	38	–	–	–
8	MPO-ANCA	>200	123	85	77	Negative	Negative	–	–
9	MPO-ANCA	Negative	Negative	Negative	Negative	Negative	–	–	–
10	MPO-ANCA	170	162	166	–	160	156	–	–
11	MPO-ANCA	Negative	Negative	Negative	Negative	Negative	Negative	–	–
12	MPO-ANCA	Negative	Negative	–	Negative	Negative	–	–	–

ANCA, antineutrophil cytoplasmic antibody; MPO, myeloperoxidase; PR3, proteinase 3.

Eight patients were ANCA positive before obinutuzumab administration. Concomitant with B-cell depletion, ANCA levels gradually declined in most (7 out of 8) patients, with 4 achieving seronegativity of ANCA. Notably, ANCA levels continued to decrease up to 1 year after obinutuzumab administration, even after B-cell reconstitution began. Only Patient 2 maintained persistently high ANCA levels despite switching to rituximab maintenance; however, her renal function remained stable.

### 3.4 Safety

Three patients experienced at least one severe adverse event after obinutuzumab therapy. Patient 3, who had progressive interstitial lung disease associated with AAV at presentation, experienced 2 separate episodes of infection with multiple pathogens, accompanied by marked thrombocytopenia. Patient 12 developed pneumonia. Both patients showed clinical improvement following antimicrobial therapy. Patient 4 experienced severe renal function deterioration due to COVID-19 infection 4 months after obinutuzumab infusion, ultimately progressing to ESKD and dialysis-dependent, but without extra-renal active lesions.

No infusion-related or allergic reaction was observed during obinutuzumab administration in these patients.

## 4 Discussion

B-cell depletion with anti-CD20 therapy has become a cornerstone of treatment for AAV. However, optimal disease control is not achieved in all patients. Obinutuzumab, a humanized type II anti-CD20 monoclonal antibody, is designed to enhance antibody-dependent cellular cytotoxicity, improve tissue penetration, and achieve deeper B-cell depletion (6–8). These properties support its potential use as an alternative anti-CD20 therapy. In a randomized trial in patients with chronic lymphocytic leukemia, obinutuzumab-based therapy was associated with higher rates of complete response and prolonged progression-free survival (9). Comparative data in primary membranous nephropathy also suggest its clinical efficacy (10). However, data of obinutuzumab in AAV is limited except for several small case series (11–14), prompting us to evaluate the efficacy and safety of obinutuzumab in AAV treatment.

In our study, 4 patients received obinutuzumab for induction therapy. Three of them achieved sustained remission, and the remaining patient progressed to ESKD due to COVID-19 infection. Notably, such sustained remission was achieved with a single 1000 mg dose of obinutuzumab. These data suggested potential efficacy at a lower dose of obinutuzumab, consistent with a recent report demonstrating remission induction using a reduced-dose regimen in refractory AAV (14), though this requires confirmation in studies of larger sample size. For maintenance therapy, 8 patients were treated with obinutuzumab and none experienced relapse during follow-up. Similarly, a prior case series of 6 rituximab-intolerant AAV patients reported only one major relapse during a 22.6-month follow-up with obinutuzumab retreatment (12). Previous studies have shown that nephrotic-range proteinuria may contribute to increased urinary loss of rituximab (22). However, evidence regarding obinutuzumab remains limited. In our study, only one patient presented with nephrotic-range proteinuria at the time of obinutuzumab administration. This patient had pre-existing impaired renal function, subsequently developed infection, and ultimately required maintenance dialysis. Therefore, the progressive renal deterioration in this case may be influenced by multiple confounding factors rather than being directly attributable to urinary loss of obinutuzumab.

Notably, in the two prior case series involving obinutuzumab (11, 12), the dosing frequency was substantially lower than with rituximab, indicating the potential for prolonged remission duration. Our data also demonstrated prolonged B-cell depletion with obinutuzumab, with B cell reconstitution typically beginning 9–12 months after the infusion. This was longer than a median of 6.1 months (IQR, 3.1–9.2) observed with rituximab in Maintenance of Remission Using Rituximab in Systemic ANCA-Associated Vasculitis (MAINRITSAN) 2 trial (16) and the ≤ 6 months depletion observed in the same patients during rituximab maintenance in our study. It should be noted that this difference may be partly attributable to the higher dose commonly used for obinutuzumab (1000 mg) compared with the 500 mg maintenance dose of rituximab in many regimens. The ongoing Obinutuzumab versus Rituximab in ANCA-associated Vasculitis (ObiVas) trial (ISRCTN13069630) will further assess whether obinutuzumab achieves deeper tissue B-cell depletion in PR3-AAV (23).

ANCA levels may help predict disease relapse, particularly after rituximab withdrawal (24). Previous trials reported that approximately half of ANCA-positive cases converted to negative following repeated rituximab treatments (16, 25), whereas emerging evidence suggests higher seroconversion rates with obinutuzumab (12). In our study, ANCA levels decreased in most patients and became undetectable in half, with the changes remaining for up to one year, which was longer than the observed period of B-cell depletion.

Several limitations of our study should be acknowledged, including its single-center, retrospective design, small sample size, and treatment heterogeneity. In particular, variations in treatment indications (induction vs. maintenance), and concomitant immunosuppressive therapies may have confounded the assessment of the independent effects of obinutuzumab. These factors restricted the generalizability and limited the ability to draw firm conclusions about long-term efficacy and safety. Nevertheless, our findings suggested that obinutuzumab is a promising treatment option for AAV, particularly in selected cases, with potential advantages in dosing frequency and durability of remission.

## 5 Conclusion

In conclusion, obinutuzumab was effective and well-tolerated in AAV patients, achieving prolonged B-cell depletion and sustained remission with reduced dosing frequency. These findings support its potential as an alternative anti-CD20 therapy for AAV and highlight its promise in optimizing long-term disease management.

## Data Availability

The original contributions presented in the study are included in the article/**Supplementary Material**. Further inquiries can be directed to the corresponding authors.
