# Supplementary material for: Obinutuzumab treatment for antineutrophil cytoplasmic antibody-associated vasculitis
Source: Front Immunol. 2026 May 13;17:1787558. doi: 10.3389/fimmu.2026.1787558 (PMC13212315; doi:10.3389/fimmu.2026.1787558)
Supplement: Supplementary file 1 [file Table1.docx]

**Supplementary Table 1. B-cell dynamics after obinutuzumab administration**

| **Patient** | **B-cell counts (cells/μL)** | | | | | | | |
| --- | --- | --- | --- | --- | --- | --- | --- | --- |
|  | **Baseline** | **Month 3** | **Month 6** | **Month 9** | **Month 12** | **Month 18** | **Month24** | **Month30** |
| 1 | 12 | 0 | 0 | 0 | 0 | - | - | - |
| 2 | - | - | - | - | 0.83 | 0 | 78 | 0 |
| 3 | - | 0 | 0 | 1 | 0 | 0 | 0 | 0 |
| 4 | 0 | 0 | - | 23 | - | - | - | - |
| 5 | 5 | 0 | 0 | 0 | 72 | - | - | - |
| 6 | - | - | - | 0 | - | - | - | - |
| 7 | 19 | 0 | 0 | 0 | 278 | - | - | - |
| 8 | 12 | 0 | 0 | 0 | 125 | 1 | 0 | - |
| 9 | 30 | 0 | 0 | 43 | 0 | 32 | - | - |
| 10 | 9 | 0 | 0 | - | 74 | - | - | - |
| 11 | 9 | 1 | 0 | 1 | 2 | 0 | - | - |
| 12 | 28 | 0 | 0 | 0 | 87 | - | - | - |
